# Effect of Temperature on Fimbrial Gene Expression and Adherence of Enteroaggregative *Escherichia coli*

**DOI:** 10.3390/ijerph120808631

**Published:** 2015-07-23

**Authors:** Woranich Hinthong, Nitaya Indrawattana, Pannamthip Pitaksajjakul, Chonlatip Pipattanaboon, Thida Kongngoen, Prapin Tharnpoophasiam, Suwalee Worakhunpiset

**Affiliations:** 1 Department of Social and Environmental Medicine, Faculty of Tropical Medicine, Mahidol University, 420/6 Ratchawithi Road, Ratchadewee, Bangkok 10400, Thailand; E-Mails: p_prem85@yahoo.com (W.H.); pannamthip.pit@mahidol.ac.th (P.P.); prapin.tha@mahidol.ac.th (P.T.); 2 Department of Microbiology and Immunology, Faculty of Tropical Medicine, Mahidol University, 420/6 Ratchawithi Road, Ratchadewee, Bangkok 10400, Thailand; E-Mails: nitaya.ind@mahidol.ac.th (N.I.); thida.kon@mahidol.ac.th (T.K.); 3 Center of Excellence for Antibody Research (CEAR), Faculty of Tropical Medicine, Mahidol University, 420/6 Ratchawithi Road, Ratchadewee, Bangkok 10400, Thailand; E-Mail: chonlatip.pip@mahidol.ac.th

**Keywords:** EAEC, temperature, aggregative adherence, biofilms, gene expression, virulence, fimbria

## Abstract

The influence of temperature on bacterial virulence has been studied worldwide from the viewpoint of climate change and global warming. The bacterium enteroaggregative *Escherichia coli* (EAEC) is the causative agent of watery diarrhea and shows an increasing incidence worldwide. Its pathogenicity is associated with the virulence factors aggregative adherence fimbria type I and II (AAFI and AAFII), encoded by *aggA* and *aafA* in EAEC strains 17-2 and 042, respectively. This study focused on the effect of temperature increases from 29 °C to 40 °C on fimbrial gene expression using real-time PCR, and on its virulence using an aggregative adherence assay and biofilm formation assay. Incubation at 32 °C caused an up-regulation in both EAEC strains 17-2 and strain 042 virulence gene expression. EAEC strain 042 cultured at temperature above 32 °C showed down-regulation of *aafA* expression except at 38 °C. Interestingly, EAEC cultured at a high temperature showed a reduced adherence to cells and an uneven biofilm formation. These results provide evidence that increases in temperature potentially affect the virulence of pathogenic EAEC, although the response varies in each strain.

## 1. Introduction

Climate change has been described as a potential threat to the global population, especially to human health, and previous research has examined the effects of climate change parameters, such as precipitation, humidity, atmospheric pressure and temperature, on particular diseases and mortality [1,2,3]. Surface water temperature was reported to be increasing both in temperate and tropical zones [4]. Temperature is known to affect the growth and virulence of bacteria [5], and some studies have also suggested a potential association between temperature and water-borne diarrheal diseases, which are a major cause of childhood mortality. The pathogens responsible for causing diarrhea are commonly found in water contaminated with fecal bacteria, such as *Salmonella* spp., *Vibrio* spp., and *Escherichia coli* (*E. coli*) [1,6,7], and a lack of sanitation and poor personal hygiene are factors responsible for increasing their infection rate.

The pathogenic enteroaggregative *E. coli* (EAEC) is the causative agent of diarrhea worldwide, especially among immunocompromised patients and children [8]. Patients exposed to EAEC from fecal-contaminated water sources can develop watery diarrhea symptoms caused by the attachment of bacteria to intestinal epithelial cells using fimbria and the release of toxins to destroy host cells [1,4,5,6,7,8]. EAEC strains demonstrate a diverse range of virulence caused by different types of virulent factors, but virulence is mainly determined by adherence ability. Currently, the gold standard for diagnosing EAEC is the Hep-2 cell adherence assay, which assesses the unique bacterial “stacked brick” pattern of adherence to cells [9]. Aggregative adherence fimbria types I and II (AAFI and AAFII), encoded by *aggA* and *aafA*, respectively, play a role in this, and are regulated by the regulator gene *aggR* [10,11,12]. *aggA* is a virulence gene of EAEC strain 17-2, while *aafA* is found in EAEC strain 042 [11,12]. Adherence fimbria are also involved in the formation of biofilm on intestinal epithelial cells, which is one of the survival and pathogenesis mechanisms of EAEC [13,14].

In 2007, the Intergovernmental Panel on Climate Change suggested that the average global temperature is predicted to increase by 1.8–4.0 °C during the 21st century [15]. This has raised concerns about human health risk from infectious waterborne diseases because temperature affects the growth and virulence of infectious pathogens. Several studies have investigated whether the influence of temperature on bacterial virulence affects disease severity, especially that of waterborne pathogens such as *E. coli*, *Vibrio coralliilyticus*, *Listeria monocytogenes* and *Salmonella* spp. [16,17,18,19]. However, information about EAEC is limited, so the present study aimed to determine the effect of temperature on adherence ability as a virulence factor of EAEC by focusing on gene expression, the capacity to attach to Hep-2 cells, and *in vitro* biofilm formation.

## 2. Materials and Methods

### 2.1. Bacterial Strains, Media, and Growth Conditions

Virulent EAEC strains 17-2 and 042 were used in this study. The bacteria were grown in Luria-Bertani (LB) broth initially at 37 °C for 24 h. They were then incubated overnight (≥18 h) in LB broth at temperatures ranging from 29 °C to 40 °C to represent predicted rises in water temperature. Bacteria were incubated with shaking to study gene expression and biofilm formation, and without shaking for the assay.

### 2.2. Virulence Gene Expression Analysis by SYBR Green Real-Time PCR

A total of 10^9^ bacterial cells cultured at various temperatures were used for RNA extraction. Whole RNA was extracted from incubated bacteria using a GeneJET RNA Purification Kit (Thermo Scientific, Waltham, MA, USA), and directly converted into cDNA by using iScript cDNA Synthesis Kit (Bio-rad, Hercules, CA, USA) via the mixture of Oligo (dT)_18_ and random hexamer primer to convert RNA into cDNA. Real-time PCR was conducted using Brilliant II SYBR^®^ Green QPCR Master Mix (Agilent Technologies, Santa Clara, CA, USA) with the following conditions: Enzyme activation at 95 °C for 10 min, then 40 cycles of denaturation at 95 °C for 30 s, annealing at 55 °C for 1 min, and melting curve analysis at 72 °C for 1 min in the Stratogene Mx3005P (Agilent Technologies, Santa Clara, CA, USA). Real-time PCR primers are listed in Table 1. EAEC strain 17-2 was used for determining the expression of *aggA*, while EAEC strain 042 was used for *aafA*. Relative mRNA levels were determined by fold-changes in expression, calculated by 2^−ΔΔCT^ using the relative mRNA level at 29 °C, representing an average water temperature, as a baseline for comparison. The results were analyzed by Mann-Whitney *U*-test using SPSS version 15.0 to determine the difference of gene expression between each temperature and 29 °C.

**Table 1:** List of primers used in the study.

Target Gene	Orientation	Sequence (5′–3′)	Amplicon Size (bp)	Reference
*16sRNA*	F	AAGTTAATACCTTTGCTCATTGAC	117	[20]
	R	GCTTTACGCCCAGTAATTCC		
*aggA*	F	CGCTGCGTTAGAAAGACCTC	164	This study
	R	CACATTGCTCTGTCGTCGTT		
*aafA*	F	ACTTCATATAGGCCTGGTCGTA	150	[21]
	R	ATTCACTCTGGCCTCTCCTAGGT		

### 2.3. Adherence Assay

Hep-2 cells were used to study the aggregative adherence ability of EAEC strains 17-2 and 042. The protocol was modified from Nataro *et al.* [9]. Cells were grown in Corning^®^ Costar^®^ 24-well treated plates (Sigma-Aldrich, St. Louis, MO, USA) in high glucose Dulbecco’s minimal essential medium (DMEM) with 10% fetal bovine serum, penicillin, and streptomycin (Hyclone Laboratories, Logan, UT, USA) at 37 °C with 5% CO_2_, until 50% confluence was reached. Cells were then replenished with 1% d-mannose DMEM to prohibit the activity of type 1 fimbria. Approximately 10^7^ cells of each EAEC strain cultured under various temperatures were fed to individual wells of cultured Hep-2 cells and incubated at 37 °C for 3 h. For qualitative Adherence assays, each well was washed three times with phosphate buffer saline (PBS), fixed with methanol, and stained with a 1:40 dilution of Giemsa in PBS. Adhesion patterns were observed under ×20 magnification using an Olympus IX71 inverted microscope (Olympus, Tokyo, Japan). In the quantitative Adherence assay, wells were washed three times with PBS then treated with 200 µL 0.1% Triton X-100 (Sigma-Aldrich) in PBS for 15 min. The detached bacteria and lysed cells then underwent serial dilution and were plated out onto LB agar to enable colony counting. Experiments were performed in duplicate. The colony forming unit mL^−1^ of each temperature was calculated for both EAEC strains 17-2 and 042. Mann-Whitney *U*-test was used to determine the difference between other temperature and 29 °C and difference between EAEC strains using SPSS version 15.0.

### 2.4. Biofilm Formation

The ability of EAEC strains 17-2 and 042 to form biofilm was determined using the biofilm-formation protocol modified from Sheikh *et al.* [22]. Bacteria were cultured in LB broth with shaking at various temperatures (29–40 °C) overnight. The cultures were adjusted to 0.5 OD600 nm, added to 24-well flat-bottomed cell culture plates containing DMEM high glucose supplemented with 1% d-mannose and incubated overnight at 37 °C. Experiments were performed in triplicate. Each well was washed three times with distilled water and stained with 0.5% crystal violet for 15 min, then washed again with distilled water and air dried. Then, 200 µL of 95% ethanol was added to each well and 150 µL of the solution transferred to 96-well flt-bottomed microplates (Sigma-Aldrich) to measure absorption at OD570 nm. Cultured samples with OD570 nm values > mean OD570 nm plus three standard deviations of the negative control (mean_neg_ + 3SD_neg_) were considered positive for biofilm formation. Differences in values between each temperature and blank were used to determine the level of biofilm formation. The data was analyzed by Mann-Whitney *U*-test using SPSS version 15.0 to test the difference between biofilm formation at 29 °C and other temperatures within EAEC strains and difference of biofilm formation between EAEC strains.

## 3. Results

### 3.1. Effects of Temperature on Virulence Gene Expression

**Figure 1:**
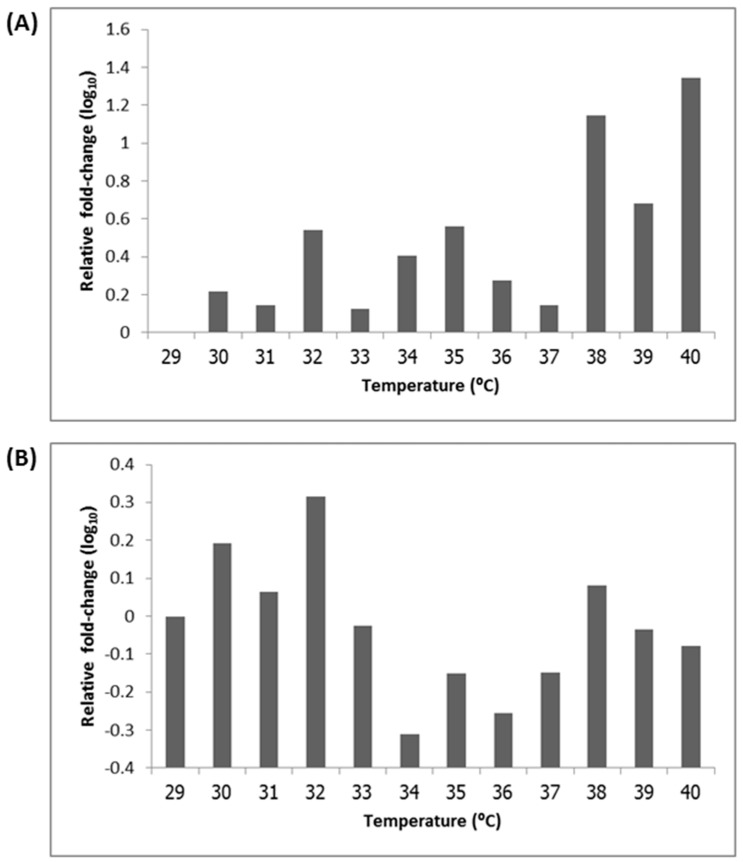
Relative mRNA levels at various temperatures (**A**) Relative *aggA* mRNA level in EAEC strain 17-2; (**B**) Relative *aafA* mRNA level in EAEC strain 042.

The expression of virulence genes (*aggA* in EAEC strain 17-2 and *aafA* in EAEC strain 042) at different temperatures was calculated with respect to *16sRNA* as a reference gene. In EAEC strain 17-2, *aggA* showed high up-regulation at 38 °C and 40 °C and relatively low expression at other experimental temperatures (Figure 1A). While in EAEC strain 042, which showed an uneven gene expression trend with the highest up-regulation at 32 °C (Figure 1B). Thus, the fimbrial genes of both EAEC strains demonstrated markedly different expression trends, although the higher expression level was observed in EAEC strain 17-2. However, the differences in gene expression at each temperature in both EAEC strains were not significantly different (*p*-value > 0.05).

### 3.2. Adherence to Hep-2 Cells

**Figure 2:**
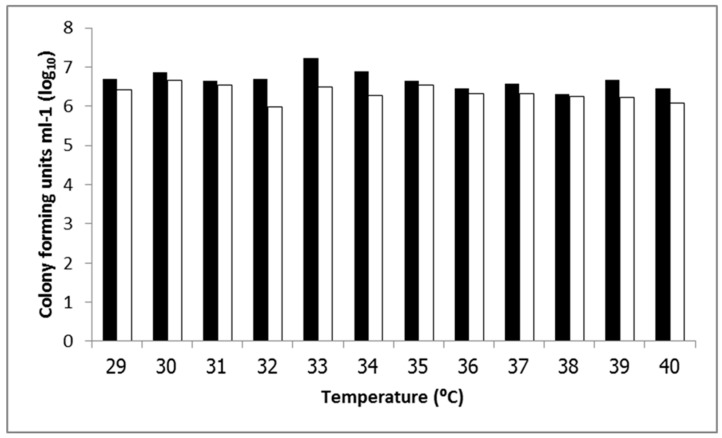
Number of colony forming units mL^−1^ from EAEC strain 17-2 (■); and strain 042 (□) in an Adherence assay.

**Figure 3:**
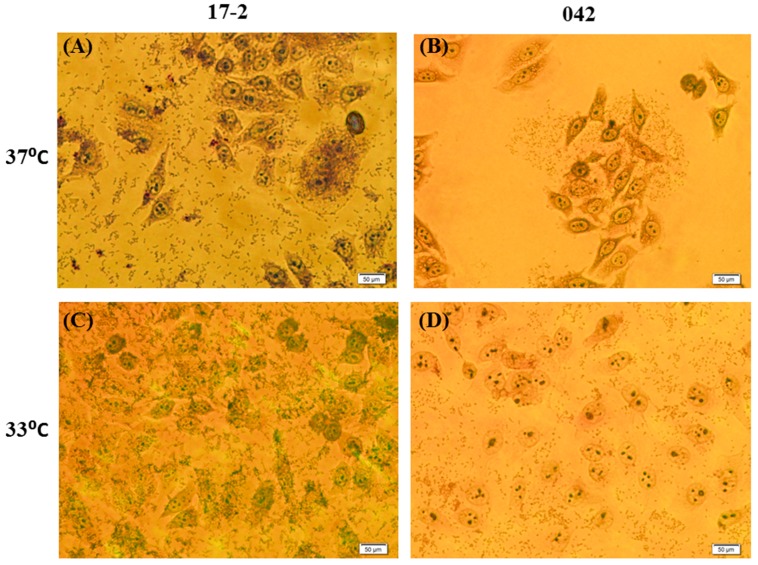
Attachment of EAEC strains 17-2 and 042 to Hep-2 cells in an Adherence assay. (**A**) 17-2 at 37 °C; (**B**) 042 at 37 °C; (**C**) 17-2 at 33 °C; and (**D**) 042 at 33 °C. Magnification ×20.

The ability of EAEC strains 17-2 and 042 to adhere to intestinal epithelial cells was determined by their ability to adhere to Hep-2 cells in an Adherence assay. For EAEC strain 17-2, the highest level of adherence was found in bacteria cultured at 33 °C and, while strain 042 showed the highest adherence at 30 °C (Figure 2). The lowest adherence of EAEC strain 17-2 was at 38 °C, and 32 °C in EAEC strain 042. At the same temperatures, a qualitative Adherence assay showed that EAEC strain 17-2 had higher numbers of bacteria adhering to Hep-2 cells than strain 042, especially at 33 °C (Figure 3). Nevertheless, the adherence between 29 °C and other temperatures and adherence of EAEC strain 17-2 and strain 042 were not significantly different (*p*-value > 0.05).

### 3.3. Biofilm Formation

**Figure 4:**
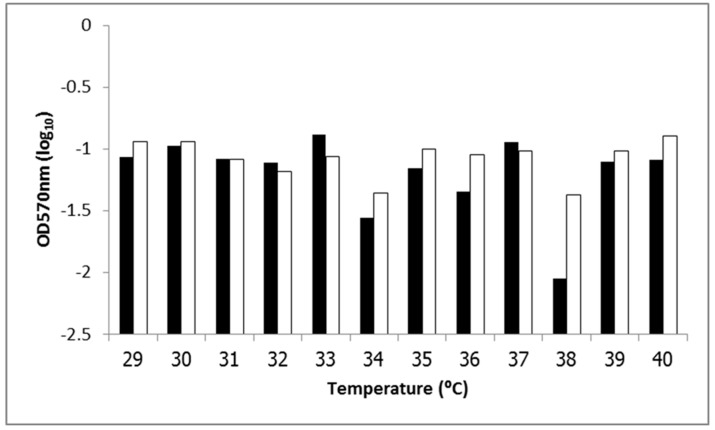
OD570 nm values from biofilm formation assay at various temperatures for EAEC strains 17-2 (■) and 042 (□).

We next studied biofilm formation to investigate the adherence ability of EAEC, and to determine whether these results confirmed those of the Adherence assay at the same temperature points. Biofilm formation by EAEC strains 17-2 and 042 under different temperatures was determined by light absorbance at OD570 nm. Although we observed a similar biofilm formation trend for both EAEC strains, EAEC strain 17-2 showed a distinct increase particularly at 33 °C which significantly different from biofilm formation at 29 °C (*p*-value < 0.05). Other temperature observed to be significantly different from biofilm formation at 29 °C of EAEC strain 17-2 included 30 °C, 32 °C, 33 °C, 34 °C, 36 °C, 37 °C, 38 °C and 40 °C. In a different manner, EAEC strain 042 showed a less distinct trend with the highest formation detected at 40 °C. But biofilm formation at 31 °C–40 °C of EAEC strain 042 was significantly different from that of 29 °C (*p*-value < 0.05). Both EAEC strains showed low biofilm formation at 34 °C and 38 °C, which represented the lowest levels for EAEC strain 042 and 17-2, respectively (Figure 4). Significant difference of biofilm formation between both EAEC strains was observed at 29 °C, 32 °C, 33 °C, 34 °C, 36 °C, 37 °C, 38 °C and 40 °C (*p*-value < 0.05).

## 4. Discussion

The effect of temperature on bacterial pathogenesis has previously been determined in *Listeria* spp., *Vibrio* spp., and *Salmonella* spp. [17,18,19]. These studies investigated many bacterial virulence factors including the adherence fimbria gene *agfD* in *Salmonella enterica* (*S. enterica*) serovar (sv) Typhimurium, *LitR* and *syp* in *Vibrio* (*Aliivibrio*) *salmonicida*, and *f9* in uropathogenic *E. coli* [23,24,25]. Morin *et al.* showed that fimbrial gene expression in EAEC strain 042 was controlled by the temperature-dependent protein H-NS, which is involved in the suppression of *aggR* expression in *E. coli* flagella biogenesis, as shown by a luciferase reporter assay [26,27]. One study in *S. enterica* sv Typhimurium reported that H-NS suppressed the expression of its virulence genes at 32 °C [28]. Similarly, our study also found low expression of *aggA* in EAEC strain 17-2 when cultured at 32 °C. However, our findings in EAEC strain 042 showed a contradiction with the study in *S. enterica* sv Typhimurium, real-time PCR results revealed *aafA* mRNA expression in EAEC strain 042 to be up-regulated at 32 °C but down-regulated at 36 °C. *aafA* in EAEC strain 042 showed a more uneven course of expression when cultured at temperatures above 32 °C. This could also be explained by the same H-NS mechanism, which was reported to regulate the switching control of *fim* at its optimum temperature (37–41 °C) in *E. coli* strain K-12 and uropathogenic *E. coli* [29,30]. Interestingly, our finding present different virulence gene expression between EAEC strain 17-2 and 042. It is possibly because the regulation of fimbrial gene expression varies between individual EAEC strains. Previous studies in *E. coli* O157:H7 also revealed that the expression of genes involving in adherence of the organism, *ehaA*, showed different expression between *in vitro* and intestinal isolates. Most of the repressed genes outside the intestine were recovered after entering the intestinal environment. Additionally, different expression behavior of *aidA*15 and *iha*, the adhesin-encoded gene, was observed between *in vivo* and *in vitro* isolates, suggesting that environmental factors potentially played a role in gene expression. Nutrition and acidity are the possible modulators influencing gene expression [31,32]. The same event was observed in *Vibrio parahaemolyticus*, which also showed different gene expression of *lafK*, which is the gene encoding for lateral flagella regulator LafK of the organism, between *in vivo* and *in vitro* [33]. Nevertheless, other environmental factors affecting virulence gene expression are yet to be reported. Our study provided the evidence supporting that temperature also distressing the adherence and virulence gene expression, which have different pattern among EAEC strains 17-2 and 042. Up- and down-regulation of virulence genes may be influenced from physiological need and stress conditions. Studies on *E. coli* regarding the adaptation to growth temperature using a microarray also indicated that *E. coli* evolved itself. Both up-regulation and down-regulation of various genes involved in high temperature response and encoded heat shock protein were observed in response to environmental conditions [34,35,36]. However, the virulence gene expression under various temperature conditions of EAEC strain 042 are not similar to other bacteria. Therefore, further study is needed to explain the possible role of H-NS in the temperature regulation of this EAEC strain 042 in which other factors may influence the expression of virulence genes. In particular, the effect of temperature on *aggR* expression should also be investigated. In the present study, we determined the adherence ability of EAEC on Hep-2 cells at various temperatures. Surprisingly, virulence gene expression of both EAEC strain, 17-2 and 042, did not appear to be correlated with Adherence assay. EAEC strain 17-2 showed the greatest adherence when cultured at 33 °C. This compares with the real-time PCR results, which demonstrated highest expression levels at 40 °C, and when cultured at 34 °C, EAEC strain 042 demonstrated its lowest *aafA* expression but elevated adherence. The cause of these phenomena may be explained by Fong *et al.*, who reported that the expression of many genes is required to achieve one phenotype [37]. Swenson *et al.* also reported that the invasive phenotype of fimbria type I of *S. enterica* sv Typhimurium was a consequence of the expression of a complex regulatory network known as the *fim* gene cluster [23]. Similarly, the adherence phenotype of EAEC relies on *aggR* as a master regulator of *aggA* and *aafA* [10,11,12,37,38]. Furthermore, Jenkins *et al.* reported the existence of chromosomal genes possibly involved in EAEC virulence that are not under the control of *aggR*, unlike plasmid-borne genes believed to be regulated by *aggR* [39]*.* Taken together, it can be implied that the adherence phenotype of EAEC involves regulation by a more complex mechanism of genes than is currently understood.

Biofilm formation is a well-known mechanism of antibiotic resistance and is involved in the pathogenesis of many bacteria. Our study found that EAEC strains 17-2 and 042 showed the highest biofilm-formation ability on an abiotic surface when cultured at 33 °C and 40 °C, respectively. Low biofilm formation was observed at 38 °C in EAEC strain 17-2, which contradicted with the gene expression result, while in EAEC strain 042 low biofilm formation result correlated with its gene expression at 34 °C. A previous study by Sheikh *et al.* (2001) reported that fimbria adhesin AAF/II of EAEC strain 042 is necessary in the stability of biofilm formation. Meanwhile, *fis* and yafK, nucleoid associated protein, also involve in the activation of biofilm formation in EAEC strain 042 [22]. This information may imply that biofilm formation of EAEC strain 042 is required the combination of many genes but lack information in strain 17-2. Moreover, possibility of new unidentified adhesins was found in several strains of EAEC [40] Therefore, the effect of temperature in the biofilm formation in EAEC strains 17-2 should be further investigated including the function of adhesin and other molecules. Although studies into the effects of temperature on biofilm formation in *E. coli* are rare, those in other bacteria, such as *Pseudomonas aeruginosa*, *Klebsiella* spp. and *Vibrio cholera* reported differences in biofilm formation ability when cultured at different temperatures, in agreement with our own findings [41]. The biofilm formation ability was correlated with virulence gene expression and adherence in EAEC strain 17-2, but not strain 042. This could be attributable to variations in the ability of bacteria to adhere to different biotic and abiotic surfaces, as well as culture times and media [24,40,41,42,43,44].

## 5. Conclusions

Our study provides evidence that temperature has an effect on EAEC adherence ability, which is one of the virulence factors of EAEC strains 17-2 and 042. The results also demonstrate variations in gene expression and phenotypes between EAEC strains, which might lead to differences in bacterial pathogenesis when affected by temperature and other factors. Because these findings are limited to EAEC possessing *aggA* and *aafA* virulence genes, and because the studies were conducted *in vitro*, further work should focus on the effects of temperature on other thermoregulator genes of EAEC and *in vivo* investigations.

## References

[1] Hashizume M., Armstrong B., Hajat S., Wagatsuma Y., Farugue A.S., Hayashi T., Sack D.A. (2007). Association between climate variability and hospital visits for non-cholera diarrhea in Bangladesh: Effects and vulnerable groups. Int. J. Epidemiol..

[2] Ou C.Q., Yang J., Ou Q.Q., Liu H.Z., Lin G.Z., Chen P.Y., Qian J., Guo Y.M. (2014). The impact of relative humidity and atmospheric pressure on mortality in Guangzhou, China. Biomed. Environ. Sci..

[3] Hashizume M., Armstrong B., Hajat S., Wagatsuma Y., Farugue A.S., Hayashi T., Sack D.A. (2008). The effect of rainfall on the incidence of cholera in Bangladesh. Epidemiology.

[4] US EPA Sea Surface Temperature. http://www.epa.gov/climatechange/science/indicators/oceans/sea-surface-temp.html.

[5] Konkel M.E., Tilly K. (2000). Temperature-regulated expression of bacterial virulence genes. Microb. Infect..

[6] Moors E., Singh T., Siderius C., Balakrishnan S., Mishra A. (2013). Climate change and waterborne diarrhoea in northern India: Impacts and adaptation strategies. Sci. Total. Environ..

[7] Singh R.B., Hales S., de Wet N., Raj R., Hearnden M., Weinstein P. (2001). The influence of climate variation and change on diarrheal disease in the Pacific Islands. Environ. Health Perspect..

[8] Kaur P., Chakraborti A., Asea A. (2010). Enteroaggregative *Escherichia coli*: An emerging enteric food borne pathogen. Interdiscip. Perspect. Infect. Dis..

[9] Nataro J.P., Kaper J.B., Robins-Browne R., Prado V., Vial P., Levine M.M. (1987). Patterns of adherence of diarrheagenic *Escherichia coli* to HEp-2 cells. Pediatr. Infect. Dis. J..

[10] Czeczulin J.R., Balepur S., Hicks S., Phillips A., Hall R., Kothary M.H., Navarro-Garcia F., Nataro J.P. (1997). Aggregative adherence fimbria II, a second fimbrial antigen mediating aggregative adherence in enteroaggregative *Escherichia coli*. Infect. Immun..

[11] Nataro J.P., Deng Y., Maneval D.R., German A.L., Martin W.C., Levine M.M. (1992). Aggregative adherence fimbriae I of enteroaggregative *Escherichia coli* mediate adherence to HEp-2 cells and hemagglutination of human erythrocytes. Infect. Immun..

[12] Nataro J.P., Deng Y., Deng Y., Walker K. (1994). *AggR*, a transcriptional activator of aggregative adherence fimbria I expression in enteroaggregative *Escherichia coli*. J. Bacteriol..

[13] Hicks S., Candy D.C., Phillips A.D. (1996). Adhesion of enteroaggregative *Escherichia coli* to pediatric intestinal mucosa *in vitro*. Infect. Immun..

[14] Pereira A.L., Silva T.N., Gomes A.C., Araujo A.C., Giugliano L.G. (2010). Diarrhea-associated biofilm formed by enteroaggregative *Escherichia coli* and aggregative *Citrobacter freundii*: A consortium mediated by putative F pili. BMC Microbiol..

[15] Solomon S., Qin D., Manning M., Chen Z., Marquis M., Tignor K.B.M., Miller H.L., IPCC (2007). Climate Change 2007: The Physical Science Basis. Contribution of Working Group I to the Fourth Assessment Report of the Intergovernmental Panel on Climate Change.

[16] Carey C.M., Kostrzynska M., Thompson S. (2009). *Escherichia coli* O157:H7 stress and virulence gene expression on Romaine lettuce using comparative real-time PCR. J. Microbiol. Methods.

[17] Leimeister-Wachter M., Domann E., Chakraborty T. (1992). The expression of virulence genes in *Listeria monocytogenes* is thermoregulated. J. Bacteriol..

[18] Kimes N.E., Grim C.J., Johnson W.R., Hasan N.A., Tall B.D., Kothary M.H., Kiss H., Munk A.C., Tapia R., Green L. (2012). Temperature regulation of virulence factors in the pathogen *Vibrio coralliilyticus*. ISME J..

[19] De Oliviera D.C., Fernandes Juniour A., Kaneno R., Silva M.G., Araujo Junior J.P., Silva N.C., Rall V.L. (2014). Ability of *Salmonella* spp. to produce biofilm is dependent of temperature and surface material. Foodborne Pathog. Dis..

[20] Chen C., Liao X., Jiang H., Zhu H., Yue L., Li S., Fang B., Liu Y. (2010). Characteristics of *Escherichia coli* biofilm production, genetic typing, drug resistance pattern and gene expression under aminoglycoside pressures. Environ. Toxicol. Pharmacol..

[21] Sharmir E.R., Warthan M., Brown S.P., Natara J.P., Guerrant R.L., Hoffman P.S. (2010). Nitazoxanide inhibits biofilm production and hemagglutination by enteroaggregative *Escherichia coli* strains by blocking assembly of *aafA* fimbriae. Antimicrob. Agents Chemother..

[22] Sheikh J., Hicks S., Dall’agnol M., Phillips A.D., Nataro J.P. (2001). Roles for *Fis* and *YafK* in biofilm formation by enteroaggregative *Escherichia coli*. Mol. Microbiol..

[23] Swenson D.L., Clegg S. (1992). Identification of ancillary fim genes affecting fimA expression in *Salmonella typhimurium*. J. Bacteriol..

[24] Hansen H., Bjelland A.M., Ronessen M., Robertson E., Willassen N.P. (2014). LitR is a repressor of *syp* genes and has a temperature-sensitive regulatory effect on biofilm formation and colony morphology in *Vibrio* (*Aliivibrio*) *salmonicida*. Appl. Environ. Microb..

[25] Wurpel D.J., Totsika M., Allsopp L.P., Hartley-Tessell L.E., Day C.J., Peters K.M., Sarkar S., Ulett G.C., Yang J., Tiralongo J. (2014). F9 fimbriae of urophathogenic *Escherichia coli* are expressed at low temperature and recognise Galβ1-3GlcNAc—Containing glycans. PLoS ONE.

[26] Morin N., Tirling C., Ivison S.M., Kaur A.P., Nataro J.P., Steiner T.S. (2010). Autoactivation of the *AggR* regulator of enteroaggregative *Escherichia coli*
*in vitro* and *in vivo*. FEMS Immunol. Med. Microbiol..

[27] Bertin P., Terao E., Lee E.H., Lejeune P., Colson C., Danchin A., Collatz E. (1994). The H-NS protein is involved in the biogenesis of flagella in *Escherichia coli*. J. Bacteriol..

[28] Ono S., Goldberg M.D., Olsson T., Esposito D., Hinton J.C., Ladbury J.E. (2005). H-NS is a part of a thermally controlled mechanism for bacterial gene regulation. Biochem. J..

[29] Gally D.L., Bogan J.A., Eisenstein B.I., Blomfield I.C. (1993). Environmental regulation of the fim switch controlling type 1 fimbrial phase variation in *Escherichia coli* K-12: Effects of temperature and media. J. Bacteriol..

[30] Kuwahara H., Myers C.J., Samoilov M.S. (2010). Temperature control of fimbriation circuit switch in uropathogenic *Escherichia coli*: Quantitative analysis via automated model abstraction. PLoS Comput. Biol..

[31] Yin X., Zhu J., Feng Y., Chambers J.R., Gong J., Gyles C.L. (2011). Differential gene expression and adherence of *Escherichia coli* O157:H7 *in vitro* and in ligated pig intestines. PLoS ONE.

[32] Yin X., Feng Y., Lu Y., Chambers J.R., Gong J., Gyles C.L. (2012). Adherence and associated virulence gene expression in acid-treated *Escherichia coli* O157:H7 *in vitro* and in ligated pig intestine. Microbiology.

[33] Livny J., Zhou X., Mandilk A., Hubbard T., Davis B.M., Waldor M.K. (2014). Comparative RNA-seq based dissection of the regulatory networks and environmental stimuli underlying *Vibrio parahaemolyticus* gene expression during infection. Nucleic Acids Res..

[34] Cases I., Lorenzo V., Ouzounis C.A. (2003). Transcription regulation and environmental adaptation in bacteria. Trends Microbiol..

[35] Riehle M.M., Bennett A.F., Lenski R.E., Long A.D. (2003). Evolutionary changes in heat-inducible gene expression in lines of *Escherichia coli* adapted to high temperature. Physiol. Genomics.

[36] Riehle M.M., Bennett A.F., Long A.D. (2005). Differential patterns of gene expression and gene complement in laboratory-evolved lines of *E. coli*. Integr. Comp. Biol..

[37] Fong S.S., Joyce A.R., Palsson B. (2005). Parallel adaptive evolution cultures of *Escherichia coli* lead to convergent growth phenotypes with different gene expression state. Genome Res..

[38] Jenkins C., Ijperon C.V., Dudley E.G., Chart H., Willshaw G.A., Cheasty T., Smith H.R., Nataro J.P. (2005). Use of a microarray to assess the distribution of plasmid and chromosomal virulence genes in strains of enteroaggregative *Escherichia coli*. FEMS Microbiol. Lett..

[39] Savarino S.J., Fox F., Deng Y., Nataro J.P. (1994). Identification and characterization of a gene cluster mediating enteroaggregative *Escherichia coli* aggregative adherence fimbria I biogenesis. J. Bacteriol..

[40] Boisen N., Struve C., Scheutz F., Krogfelt K.A., Nataro J.P. (2008). New adhesin of enteroaggregative *Escherichia coli* related to the Afa/Dr/AAF family. Infect. Immun..

[41] Hostacka A., Ciznar I., Stefkovicova M. (2010). Temperature and pH affect the production of bacterial biofilm. Folia Microbiol..

[42] Sakyi P.A., Asare R. (2012). Impact of temperature on bacterial growth and survival in drinking-water pipes. Res. J. Environ. Earth Sci..

[43] Reisner A., Krogfelt K.A., Klein B.M., Zechner E.L., Molin S. (2006). *In vitro* biofilm formation of commensal and pathogenic *Escherichia coli* strains: Impact of environmental and genetic factors. J. Bacteriol..

[44] Uhlich G.A., Chen C.Y., Cottrell B.J., Nguyen L. (2014). Growth media and temperature effects on biofilm formation by serotype O157:H7 and non-O157 shiga toxin-producing *Escherichia coli*. FEMS Microbiol..

